# Highly Selective and Ultrasensitive Turn-On Luminescence Chemosensor for Mercury (II) Determination Based on the Rhodamine 6G Derivative FC1 and Au Nanoparticles

**DOI:** 10.3390/s16101652

**Published:** 2016-10-06

**Authors:** Romina Brasca, María C. Onaindia, Héctor C. Goicoechea, Arsenio Muñoz de la Peña, María J. Culzoni

**Affiliations:** 1 Laboratorio de Desarrollo Analítico y Quimiometría (LADAQ), Universidad Nacional del Litoral, CONICET, FBCB, Ciudad Universitaria, Santa Fe 3000, Argentine; rbrasca@fiq.unl.edu.ar (R.B.); celeste.onaindia@gmail.com (M.C.O); hgoico@fbcb.unl.edu.ar (H.C.G); 2 Department of Analytical Chemistry and IACYS, University of Extremadura, Badajoz 06006, Spain

**Keywords:** rhodamine 6G derivative, Au nanoparticles, Hg^2+^ chemosensor, fluorimetric assay, aqueous solution

## Abstract

A method for the detection and quantitation of Hg^2+^ in aqueous samples by fluorescence spectroscopy is presented. It consists of a turn-on sensor developed by coupling Gold nanoparticles (AuNPs) with the rhodamine 6G derivative FC1, in which the response is generated by a mercury-induced ring-opening reaction. The AuNPs were included in order to improve the sensitivity of the method towards the analyte, maintaining its high selectivity. The method was validated in terms of linearity, precision and accuracy, and applied to the quantitation of Hg^2+^ in Milli-Q and tap water with and without spiked analyte. The limit of detection and quantitation were 0.15 μg·L^−1^ and 0.43 μg·L^−1^, respectively, constituting a substantial improvement of sensitivity in comparison with the previously reported detection of Hg^2+^ with free FC1.

## 1. Introduction

Environmentally, mercury contamination of ecosystems occurs through a variety of natural and anthropogenic sources. These sources include oceanic and volcanic emissions, forest fires, gold mining, solid waste incineration, and the combustion of fossil fuels, resulting in increasing levels of Hg^2+^ emissions into the air, soil and water. Inorganic mercury can cause a wide range of diseases such as digestive, heart, kidney and especially neurological disorders [1]. Mercury is one of the most harmful pollutants because it can easily pass through skin, respiratory, and gastrointestinal systems. A major absorption source is related to daily food such as fish. Therefore, it is important to monitor Hg^2+^ levels in aquatic ecosystems as a potential source of contamination, since the situation becomes increasingly serious to the living environment of humans and other species. The United States Environmental Protection Agency (US-EPA) has set the safety level of Hg^2+^ concentration for drinking water at 10 nM (2 μg·L^−1^) [2], and the World Health Organization [3] and the European Union [4] indicate for this ion a value of 5 nM (1 μg·L^−1^), highlighting the necessity of developing sensitive methods for its determination. 

Many current techniques for mercury determination, such as atomic absorption/emission spectroscopy, inductively coupled plasma mass spectrometry, and selective cold vapor atomic fluorescence spectrometry, require sophisticated instrumentation and/or complicated manipulations and time-consuming sample preparation processes [5]. In this context, considerable attention has recently been focused on the exploration of simple and fast detection methods for aqueous Hg^2+^ ions and, particularly, on the design of luminescence chemodosimeters due to the highly sensitive, quick, and nondestructive advantages of luminescence methods, which make them an ideal probe displaying very low detection limits and high selectivity towards Hg^2+^ in the presence of other metals [6,7].

The chemodosimeter strategy is based on the use of a selective reaction that is induced by the target species and gives rise to an observable luminescence signal. Generally, Hg^2+^ ions are known to produce fluorescence quenching when binding to fluorophore molecules via the spin-orbit coupling effect. However, a turn-on response is preferable, since the ubiquitous nature of fluorescence quenching reduces to some extend the practical utility of the turn-off probes. Therefore, fluorescent turn-on type molecular probes for monitoring the level of Hg^2+^ in environmental and biological samples is of current interest, and rhodamine and boron-dipyrromethene (BODIPY) derivatives are examples of families of small molecular probes successfully reported for selective turn-on Hg^2+^ detection [8,9].

Rhodamine 6G spirocyclic phenylthiosemicarbazide derivative (FC1) has been previously proposed for Hg^2+^ determination in diverse matrices, such as water and fish samples [10,11,12], reporting a limit of detection of 1 μg·L^−1^ [10]. Unlike other chemosensors, the probe responds to Hg^2+^ in an irreversible manner, as the rhodamine 6G derivative undergoes oxadiazole formation when the thiosemicarbazide moiety is liberated by Hg^2+^, facilitating ring opening of the spirocycle group. The reaction is based on the well-known spirolactam (nonfluorescent) to ring opened amide (fluorescent) equilibrium of rhodamine derivatives [13,14,15], coupled to the irreversible Hg^2+^-promoted reaction of thiosemicarbazides to form 1,3,4-oxadiazoles [16,17]. The same probe and mechanism were also used in a LED excited portable fiber-optic system, lowering the limit of detection to 0.7 μg·L^−1^ [18]. The probe has been also proposed for methylmercury fluorescent detection [19].

It is interesting to point out that, although the number of examples of rhodamine derivative probes reported for Hg^2+^ detection is high, there are only few examples of probes immobilized in solid supports [20,21,22] and, in an effort to develop a probe for the construction of an optosensor, several approaches have been explored to immobilize FC1 in a solid support. In this sense, the procedure was improved by firstly immobilizing the reagent in a nylon membrane [23,24]. With this approach, the limit of detection of the method was lowered to 0.4 μg·L^−1^, and the probe was applied to the determination of Hg^2+^ residues in drinking and environmental waters. Later on, the probe was immobilized on hydrophilic water-insoluble poly(2-hydroxyethylmethacrylate-co-methylmethacrylate) co-polymer [25], and encapsulated in electrospinning generated microfiber nonwoven mats [26]. Then, the developed sensing films was applied to the detection of mercury and methylmercury ions in tap and mineral waters, although in these two approaches, the detection limit was higher than in solution.

AuNPs with well-defined structures have become an active research area, and combination of organic chromophores and AuNPs for producing typical organic/inorganic hybrid materials and unique photophysical and photochemical properties is still a challenge. Interestingly, they are excellent quenchers of organic fluorophores via resonance energy transfer (RET) and can efficiently quench the molecular-excitation energy in the chromophore–AuNP composite [27]. In this sense, the quenching property of AuNPs has been employed in several chemosensing schemes [28,29], and many approaches have been used for Hg^2+^ determination using AuNPs, being an area of current interest [30,31,32,33,34,35,36]. Usually, the RET process occurring in a bimolecular dye-quencher system through single dipole–dipole interaction is the Förster RET (FRET) [37]. Some authors postulate that, in the case of AuNPs, the dipole–dipole interaction is replaced by a dipole–multipole interaction due to the metallic surface, and the process is known as nanomaterial surface energy transfer (NSET) [38]. In the case of rhodamine–AuNPs systems, different studies suggested the occurrence of FRET [32,39], NSET [40,41], and both FRET and NSET simultaneously [42]. It is important to mention that the partial or complete overlap between the SPR band of the AuNPs and the emission band of the dye, acting in this case the rhodamine as the donor and the AuNPs as the acceptor, is a prerequisite for the occurrence of the efficient energy transfer.

Therefore, it is expected that in the presence of AuNPs FC1 exhibits lower fluorescence than in its absence (i.e., free FC1) due to the RET effect. In this paper, we report on the use of this effect for enhancing the sensitivity of the former method based on the reaction of FC1 towards Hg^2+^, maintaining its high selectivity.

## 2. Material and Methods

### 2.1. Instruments

Fluorescence measurements were carried out on a Perkin Elmer LS 55 luminescence spectrometer (Llantrisant, UK) equipped with a xenon discharge lamp, using a 1.00 cm quartz cell and 10 nm of excitation and emission slit widths. The photomultiplier tube voltage was set to 800 V. The emission spectra were recorded between 540 and 600 nm at the excitation wavelength of 528 nm, with the exception of some cases mentioned below. The fluorescence measurements were made using a thermostated cell holder and a thermostatic bath (ORL Hornos Eléctricos S.A., Buenos Aires, Argentina). Absorbance measurements between 200 and 800 nm were obtained using a 1.00 cm quartz cell with a Perkin Elmer Lambda 20 spectrophotometer (Waltham, MA, USA).

The pH of solutions was measured with an Orion 410 A potentiometer (Beverly, MA, United States) equipped with a Boeco BA 17 (Hamburg, Germany) combined glass electrode. 

Measurements of average diameter and size distribution of AuNPs were performed using a Zetasizer Nano S90 Malvern Dynamic Light Scattering (DLS) instrument (Malvern, UK).

### 2.2. Reagents and Procedures

Analytical reagent-grade chemicals and ultrapure water, obtained from a Milli-Q water purification system from Millipore (Kankakee, IL, USA), were used throughout the experiments. HPLC-grade MeOH and ACN were purchased from Merck Millipore (Danvers, MA, USA).

FC1 was synthesized following the procedure previously described in the literature [10,12,18]. The stock solution of FC1 1 mmol·L^−1^ was prepared by dissolving 5.0 mg of FC1 in 10.00 mL of ACN. This solution was further diluted by 100 times in water:MeOH (80:20, pH = 7) to prepare an intermediate solution, which was kept in the fridge before being used. 

AuNPs were synthesized by chemical reduction according to the procedure reported by Turkevich-Frens [43,44] utilizing aqueous solutions of AuCl_3_ 15.0 g·L^−1^ and sodium citrate 50.0 g·L^−1^. Using Beer’s law, the concentration of the citrate-capped AuNPs solution was estimated to be 5.17 × 10^−8^ mol·L^−1^ (absorption at 520 nm and an extinction coefficient of 3.67 × 10^−8^ L·mol^−1^·cm^−1^ for particles of 15 nm diameter were used for the calculations [45]). The as-prepared AuNPs solution was diluted into solutions of different concentrations for subsequent characterization, i.e., a 1.29 × 10^−9^ mol·L^−1^ solution in Milli-Q water for the registration of the UV-Vis spectrum between 400 and 800 nm, and an 8.61 × 10^−9^ mol·L^−1^ solution for DLS measurements.

The stock solution of HgCl_2_ (2.173 g·L^−1^) was prepared by dissolving 217.3 mg of HgCl_2_ in 100.00 mL of Milli-Q water containing a few drops of concentrated HNO_3_ and further diluted whenever necessary. 

In order to perform the interference study with metal cations, several 2.00 μg·L^−1^ stock solutions were prepared by weighing and dissolving appropriate quantities of each salt in Milli-Q water: Li_2_CO_3_, NaCl, KCl, AgNO_3_, MgCl_2_, CaCl_2_, BaCl_2_, FeSO_4_, CuCl_2_, ZnSO_4_, Cd(CH_3_COO)_2_, CoSO_4_·7H_2_O, Pb(NO_3_)_2_, Fe_2_(SO_4_)_3_ and Al_2_(SO_4_)_3_. All stock solutions were stored in amber glass bottles at 4 °C.

### 2.3. Sensor Development and Characterization

In order to qualitatively evaluate the interaction between FC1 and AuNPs in terms of the occurring energy transfer process, the emission spectrum of a FC1 solution 1 μmol·L^−1^ and the UV spectrum of a AuNPs solution 1.29 × 10^−9^ mol·L^−1^, both prepared in water:MeOH (80:20, pH = 7), were recorded and graphically overlapped.

With the purpose of establishing the quantity of AuNPs needed to suppress the signal attributable to FC1, increasing volumes between 5.0 and 140.0 μL of the AuNPs stock solution were added to 2.00 mL of a FC1 solution 1 μmol·L^−1^ prepared in water:MeOH (80:20, pH = 7), and the emission fluorescence spectrum was recorded after each addition. In order to calculate the energy transfer efficiency, an emission spectrum of a solution having 250.0 μL of the FC1–AuNPs sensor, i.e., 200.0 μL of FC1 10 μmol·L^−1^ and 50.0 μL of AuNPs stock solution in 2.00 mL of water:MeOH (80:20, pH = 7), was registered and, then, the FC1–AuNPs emission spectrum was compared with the emission spectrum of a FC1 solution 1 μmol·L^−1^.

Besides, in order to evaluate the influence of the Hg^2+^ concentration on the sensor response, a solution containing 250.0 μL of the FC1–AuNPs sensor in 2.00 mL of water:MeOH (80:20, pH = 7) was prepared and transferred to a quartz cuvette. Then, 5.0 μL aliquots of Hg^2+^ solutions of several concentrations (0.4, 4.0, 20.0 and 40.0 mg·L^−1^) were added to gradually increment the Hg^2+^ concentration before recording each fluorescence spectrum. 

The stability of the sensor response in the presence of Hg^2+^ was studied by adding 250.0 μL of the FC1–AuNPs sensor into a 2.00 mL volumetric flask and completed to the mark with water:MeOH (80:20, pH = 7). After homogenizing, the solution was spiked with Hg^2+^ in order to obtain a final Hg^2+^ concentration of 5.00 μg·L^−1^ and the emission spectra were registered every 30 min, during 480 min, at the working temperature of 40 °C. 

The influence of the organic solvent was evaluated by substituting different volumes of water for MeOH in 2.00 mL solutions having 250.0 μL of the FC1–AuNPs sensor and completed to the mark with a solution having 2.00 μg·L^−1^ of Hg^2+^ in order to reach percentages of MeOH ranging from 0% to 20% each 5% intervals. The experiment was performed in triplicate and the fluorescence emission spectra were registered.

An interference study was performed to gather information regarding the selectivity of the FC1–AuNPs sensor towards Hg^2+^. In order to evaluate different metallic ions, i.e., Li^+^, Na^+^, K^+^, Ag^+^, Mg^2+^, Ca^2+^, Ba^2+^, Fe^2+^, Cu^2+^, Zn^2+^, Cd^2+^, Co^2+^, Pb^2+^, Fe^3+^ and Al^3+^, several 2.00 mL solutions containing 250.0 μL of FC1–AuNPs sensor and completed to the mark with solutions containing 2.00 μg·L^−1^ of each metallic ion dissolved in water:MeOH (80:20, pH = 7) were prepared and monitored in the established conditions.

It is important to mention that the pH was set to 7 in every experiment, as the sensor is sensitive to pH. This behavior was firstly studied and reported by Yang and coworkers [10], and further confirmed by Bohoyo and coworkers [18]. These latter authors informed that pH-controlled emission measurements showed that FC1 responds to Hg^2+^ in the pH range from 5.5 to 12.0 with the fluorescent intensity varying less than 10%. When the pH value is lower than 5.0, the luminescence intensity of free FC1 increases greatly with decreasing pH values, and then a value of pH = 7.0 was selected as optimum.

All the experiments were carried out in the darkness. 

### 2.4. Method Validation: Limit of Detection (LOD), Limit of Quantitation (LOQ), Linearity, Precision and Accuracy

The proposed method was validated in order to test its performance regarding linearity, precision and accuracy.

In order to calibrate, six Hg^2+^ solutions having 0.00, 0.50, 1.00, 1.50, 2.00 and 3.00 μg·L^−1^ in Milli-Q water were prepared in triplicate by adding appropriate amounts of Hg^2+^ stock solution into 10.00 mL volumetric flasks. Then, 1600.0 μL of each standard was mixed with 400.0 μL of MeOH and the pH was adjusted to 7.00 with NaOH 0.1 mol·L^−1^. Finally, 250.0 μL of FC1–AuNPs sensor were added to 2.00 mL volumetric flasks, completed to the mark with each corresponding standard solutions and incubated at 40 °C during 7 h before registering the emission spectra. 

The method precision was estimated by preparing and analyzing 10 replicates of samples having 2.00 μg·L^−1^ of Hg^2+^ in Milli-Q water during three consecutive weeks. Both interassay and intermediate precisions were estimated by performing an ANOVA test.

Finally, with the aim of evaluating accuracy and testing the applicability of the method both in Milli-Q water and tap water from Santa Fe city, appropriate aliquots of Hg^2+^ stock solution were added to 10.00 mL volumetric flasks in order to have three Hg^2+^ concentration levels: 1.00, 1.50 and 2.00 μg·L^−1^. The samples were processed in triplicate according to the proposed method, and before analyzing, they were filtered through a membrane filter of 0.45 μm particle size.

## 3. Results and Discussion

### 3.1. Sensor Development and Characterization

**Figure 1:**
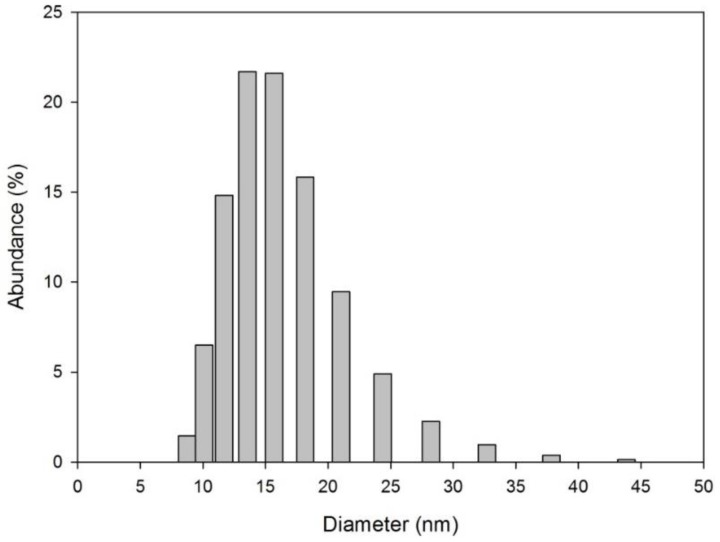
DLS particle size distribution of monodisperse AuNPs with an average size of 15 nm.

According to the size distribution plot achieved by DLS measurements for the synthesized AuNPs shown in Figure 1, monodisperse AuNPs of ca. 15 nm were obtained. 

**Figure 2:**
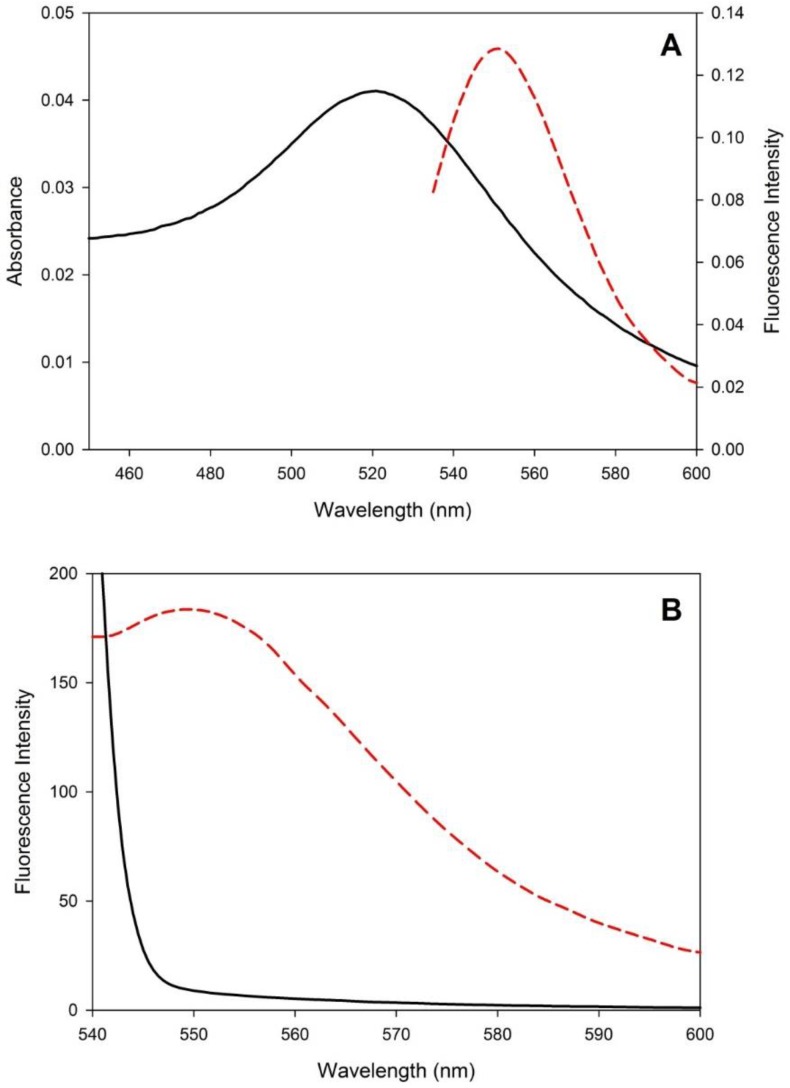
(**A**) Normalized absorption spectrum of a solution of AuNPs 1.29 × 10^−9^ mol·L^−1^ (black solid line) and normalized emission spectrum (*λ*_ex_ = 528 nm) of a solution of FC1 1 μmol·L^−1^ (red dashed line), both solutions prepared in water:MeOH (80:20, pH = 7); and (**B**) fluorescence emission spectra of a solution of FC1 1 μmol·L^−1^ before (red dashed line) and after (black solid line) the addition of 50 μL of AuNPs 5.17 × 10^−8^ mol·L^−1^ (*λ*_ex_ = 528 nm).

With the purpose of diminishing the fluorescent basal signal of FC1 taking advantage of the RET process as a strategy to increase the sensor sensibility towards the detection of Hg^2+^ ions, the feasibility of coupling it to AuNPs was investigated. Firstly, the overlap degree between the absorption spectrum of the synthesized AuNPs (1.29 × 10^−9^ mol·L^−1^) and the emission spectrum of FC1 (1 μmol·L^−1^) was analyzed. As can be seen in Figure 2A, the FC1 emission spectrum is completely overlapped with part of the AuNPs absorption spectrum, a fact that supports the ultrafast and effective energy transfer. Then, modifications in the fluorescence emission spectrum of the FC1 solution 1 μmol·L^−1^ due to the presence of different concentrations of AuNPs were evaluated to determine the suitable amount of AuNPs stock solution needed to deactivate its basal emission. According to this experiment, the free FC1 basal emission fluorescence signal is almost completely quenched in the presence of 50.0 μL of the AuNPs stock solution (Figure 2B), and there is no need of centrifuging the samples before registering the fluorescence emission spectra.

The very low fluorescence emission observed for the FC1–AuNPs solution suggests that the FC1 molecules had been effectively adsorbed onto the AuNPs surface, and that their fluorescence emission was strongly quenched by the AuNPs via a RET process. The transfer efficiency was calculated as $=1-\frac{F}{F_{0}}$, where *F* is the donor fluorescence intensity in the presence of the acceptor, and *F*_0_ is the donor fluorescence intensity in the absence of the acceptor [46], and a value of 0.97, indicative of the process efficiency, was obtained. 

As mentioned in the Introduction, several authors suggested the occurrence of a RET and collision process between the rhodamine and AuNPs [32,39], and it is also known that this process is usually occurring in a biomolecular dye-quencher system through single dipole–dipole interaction FRET [37]. Other authors suggested that in the case of AuNPs the dipole–dipole interaction is replaced by a dipole–multipole interaction due to the metallic surface, and the process is known as NSET [40,41], or that these two mentioned processes, i.e., FRET and NSET, could occur simultaneously [42].

Consistent with the previous results, the optimum composition of the FC1–AuNPs sensor was established as 200.0 μL of FC1 10 μmol·L^−1^ and 50.0 μL de AuNPs 5.17 × 10^−8^ mol·L^−1^, to be mixed with 1750.0 μL of aqueous sample:MeOH (80:20, pH = 7). In these conditions, the final concentrations of FC1 and AuNPs were 1 μmol·L^−1^ and 1.29 × 10^−9^ mol·L^−1^, respectively, with a FC1:AuNPs molar relationship of 774:1. It is important to mention that in the sensor the interaction between FC1 and AuNPs is just by mixing them until the solution is completely homogenized, i.e., by the direct contact of the FC1 molecule with the metal AuNPs surface.

**Figure 3:**
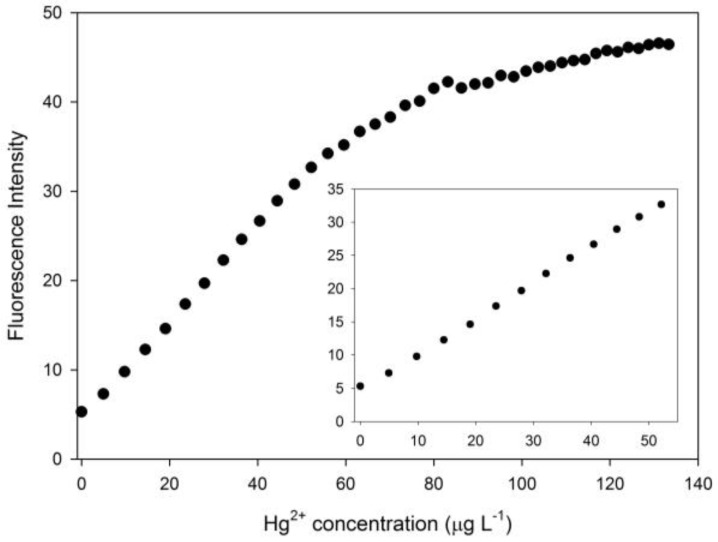
Fluorescence emission variations at 555 nm (*λ*_ex_ = 528 nm) of the sensor in the presence of increasing Hg^2+^ concentrations. The linear region at low Hg^2+^ concentrations is amplified in the inset.

The following step consisted in evaluating the sensor response in the presence of Hg^2+^. Figure 3 illustrates the complete preliminary titration conducted by registering the fluorescence emission intensity variation at 555 nm as long as the Hg^2+^ concentration increases until the fluorescence signal development due to Hg^2+^ tends to reach the plateau (i.e., 140 µg·L^−1^). The evidenced linear relationship between fluorescence emission and Hg^2+^ concentration at low Hg^2+^ concentrations, which is amplified in the inset of Figure 3, supported the idea of developing a simple analytical method to satisfactorily detect extremely low Hg^2+^ levels in aqueous matrices due to the promising quantitative capabilities of the sensor regarding basal signal suppression and subsequent enhancement due to a chemical reaction. 

### 3.2. Optimization of the Chemodosimeter Response 

Based on the previous experiments, a more in-depth study of the different experimental parameters that may have influence on the sensor response towards Hg^2+^ was conducted. 

The experiment involving the registration of the emission spectra each 30 min during 480 min after starting the reaction between FC1–AuNPs and 5.00 μg·L^−1^ of Hg^2+^ showed that the fluorescence emission intensity starts to stabilize after increasing during 420 min. According to this result, the reaction time was set to 7 h under incubation at 40 °C. It is worth mentioning that current experiments are being conducted in our laboratory in order to reduce the incubation time with the purpose of improving the efficiency of the proposed method.

Given that the presence of 20% organic solvent in the reaction medium has an effect on the free FC1 fluorescence intensity response in the presence of Hg^2+^ [18], the influence of MeOH was evaluated for the FC1–AuNPs sensor. It was confirmed that the best fluorescence signal was achieved in the presence of 20% of organic solvent, and that the fluorescence response due to Hg^2+^ is negligible in the absence of MeOH.

**Figure 4:**
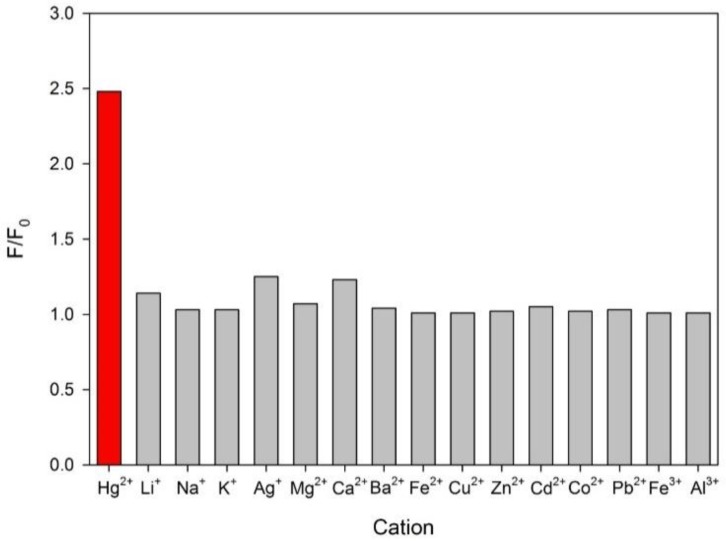
Bars representing the final (F) over the initial fluorescence emission of the probe (F_0_) in the presence of 2.00 μg·L^−1^ of several cations.

The selectivity of the FC1–AuNPs sensor towards Hg^2+^ ions was assessed by testing the fluorescence response in the presence of a great variety of metallic cations under identical experimental conditions. Figure 4 demonstrates that the sensor practically does not react to 2.00 μg·L^−1^ of the assessed cations, compared to the signal obtained for 2.00 μg·L^−1^ of Hg^2+^, a fact that allows concluding about its ability to selectively detect Hg^2+^ in aqueous samples that also contain several metallic cations, without having to perform tedious samples cleanup. The results obtained in our selectivity study are in agreement with the ones obtained in previous publications with similar rhodamine or fluorescein derivatives based on the same irreversible desulfuration reaction to form the corresponding oxodiazole. See, for example, the following work [47], where in the performed interference study in the presence of other metal ions as Fe^2+^, Co^2+^, Ni^2+^, Cu^2+^, Zn^2+^, Mn^2+^, Ag^+^, Cd^2+^, Pb^2+^, K^+^ and Mg^2+^, it was stated that “because the K_sp_ value of HgS is the lowest among the tested metal ions, the desulfurization reaction triggered by Hg^2+^ is the most efficient. As a result, the selectivity of the reagent toward Hg^2+^ ions over other metal ions is remarkably high”. Furthermore, since the sensor has been thought to be applied to the determination of Hg^2+^ in real water samples, which have a great variety not only of cations but also of anions, the study becomes more representative of the reality when it is conducted in the presence of different anions.

### 3.3. Analytical Parameters and Applications

The statistical parameters obtained by least-squares fitting of the calibration curve for Hg^2+^ are summarized in Table 1. It should be pointed out that the method sensitivity in terms of both LOD and LOQ, calculated according to [48], has been improved with respect to those previously documented in the literature for Hg^2+^ detection using free FC1. In this sense, in the first work, which was mainly related to its development and characterization, the authors stated that the chemosensor allows detecting 1 μg·L^−1^ of Hg^2+^ [10]. Subsequently, Bohoyo et al. reported a LOD of 0.7 μg·L^−1^ for Hg^2+^ detection in water samples [18]. The herein proposed methodology allows detecting Hg^2+^ at 0.15 μg·L^−1^, which is in agreement with the requirements imposed by national and international norms, and constitutes a substantial improvement with respect to the previously quoted publications. There are other reports achieving very low detection limits for Hg^2+^ in aqueous solution as can be seen, for example, in a comparative study published in Table 2 of [39], comparing the linear range and detection limit of nine selected fluorimetric methods for Hg^2+^ detection, including those reported in [31,32,39]. As already stated, the LOD of the proposed method in the present manuscript is 0.15 µg·L^−1^, which corresponds to 0.75 × 10^−9^ M. The LODs reported for the gold nanoparticles based rhodamine derivative reagents in [31,32,39] are 10 × 10^−9^ M, 0.06 × 10^−9^ M and 0.6 × 10^−9^ M, respectively. Our method favorably compares with that of [31] and is practically comparable to that reported in [39]. All other reagents reported in the mentioned table are higher than the LOD of the present method.

Table 2 shows the results obtained for the precision study. As can be seen, the ANOVA analysis for the results provided by the proposed methodology for 10 replicates of the same sample during three consecutive weeks allows concluding about the method precision with a significance of 95%. Besides, both inter and intra assay precision are comparable since *p* is higher than 0.05. 

**Figure 5:**
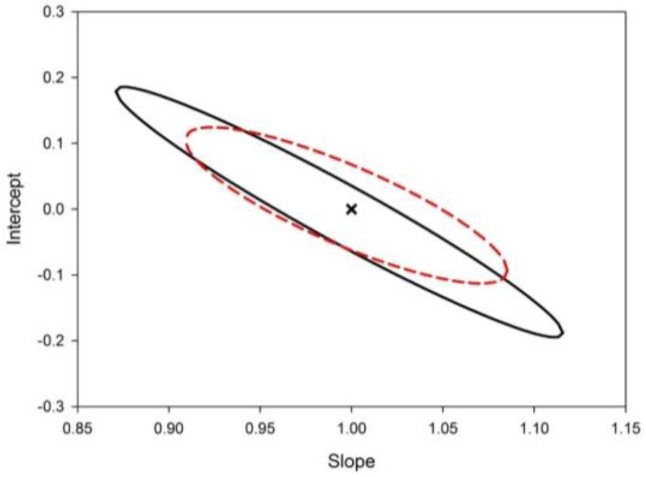
Elliptical joint confidence regions for the slope (b) and intercept (a) corresponding to a linear regression of nominal concentrations of accuracy samples vs. Hg^2+^ concentrations found for each analyzed matrix, i.e., Milli-Q water (black solid line) and tap water (red dashed line). The black cross indicates the theoretical point (a = 0, b = 1).

With the purpose of testing the capability of the analytical method to correctly quantitate Hg^2+^ in aqueous matrices, both Milli-Q water and tap water from Santa Fe city were spiked with three concentration levels of the analyte and analyzed by triplicate. Table 3 shows the prediction results with recovery percentages near to 100% for all the samples. Figure 5 illustrates the elliptical joint confidence region (EJCR, [49]) test for the slope and intercept of the plots corresponding to nominal versus predicted Hg^2+^ concentration for each analyzed matrix. Both ellipses include the theoretically expected values of (1, 0) for slope and intercept, respectively, indicating that both systematic and proportional errors are absent. In light of this evidence, it can be stated that the proposed methodology is suitable for the quantitation of Hg^2+^ in this kind of matrices.

## 4. Conclusions

In conclusion, an analytical method that allows detecting and quantitating Hg^2+^ in aqueous samples by fluorescence spectroscopy has been developed and validated. The FC1–AuNPs sensor proved to be selective for Hg^2+^ and provides lower limit of detection and quantification, i.e., 0.15 μg·L^−1^ and 0.43 μg·L^−1^, respectively, than those previously reported in the literature, constituting a substantial improvement regarding sensitivity in the detection of Hg^2+^. The validation results concerning linearity, precision and accuracy endorses the methodology for the evaluation of Hg^2+^ in aqueous samples in the sub-part per billion level. Therefore, the proposed methodology is adequate to detect and quantitate Hg^2+^ in drinking and tap water samples, in agreement with the requirements imposed by national and international norms.

## Figures and Tables

**Table 1:** Analytical figures of merit.

Figure of Merit	Value
Intercept (SD) ^a^	7.0 (0.2)
Slope (L·μg^−1^) (SD) ^a^	7.2 (0.1)
R	0.9983
Analytical sensitivity (γ) (L·μg^−1^)	16.4
γ^−1^ (μg·L^−1^)	0.06
LOD (μg·L^−1^) ^b^	0.15
LOQ (μg·L^−1^) ^b^	0.43
Linearity range (μg·L^−1^)	0.43–3.00
*F*_exp_^c^	0.77

^a^ SD: Standard deviation for n−1 degrees of freedom. ^b^ LOD (limit of detection) and LOQ (limit of quantification) calculated according to [48]. ^c^ *F*-test for linearity determination. *F*_tab(18-2);(18-6);0.05_ value equal to 2.60.

**Table 2:** Results obtained for the precision set, coefficient of variation and ANOVA probability.

Parameter	Result
Week 1 (SD) ^a^	1.50 (0.03)
Week 2 (SD) ^a^	1.53 (0.04)
Week 3 (SD) ^a^	1.51 (0.05)
Intermediate precision (CV%) ^b^	2.94
ANOVA ^c^	*F* = 2.64 (*p* = 0.08)

^a^ SD: Standard deviation for n−1 degrees of freedom for ten replicates. ^b^ CV: Percentage variation coefficient; CV% = (SD/average concentration) × 100. ^c^ *F*_tab(3-1);(30-3);0.05_ value equal to 3.35.

**Table 3:** Recovery study of Hg^2+^ in spiked water samples.

Sample	Hg^2+^ Concentration (μg·L^−1^)	
	Nominal	Found ^a^
Milli-Q	1.00	0.99 (0.01)[99.0]
	1.50	1.49 (0.03)[99.1]
	2.00	1.99 (0.07)[99.3]
Tap water ^b^	0.00	ND ^c^
	1.00	0.99 (0.04)[99.3]
	1.50	1.48 (0.08)[98.7]
	2.00	2.0 (0.1)[101.2]

^a^ Experimental standard deviation of triplicates, in the last significant figure, in parentheses. The recoveries (in square brackets) are based on the added amounts. ^b^ From Santa Fe city (Santa Fe, Argentina). ^c^ ND: Not detectable.
